# Supplementary material for: Time‑resolved multi-omic analysis of paclitaxel exposure in human iPSC‑derived sensory neurons unveils mechanisms of chemotherapy‑induced peripheral neuropathy
Source: Cell Death Dis. 2026 Feb 10;17(1):211. doi: 10.1038/s41419-026-08445-2 (PMC12921266; doi:10.1038/s41419-026-08445-2)
Supplement: Supplementary file 15 — Dataset 1. [file 41419_2026_8445_MOESM15_ESM.zip › CDD_Open_Data/05_Protein/02_Intersects_Disco_Plots/Andranik/48h.RNA_vs_protein.pdf]

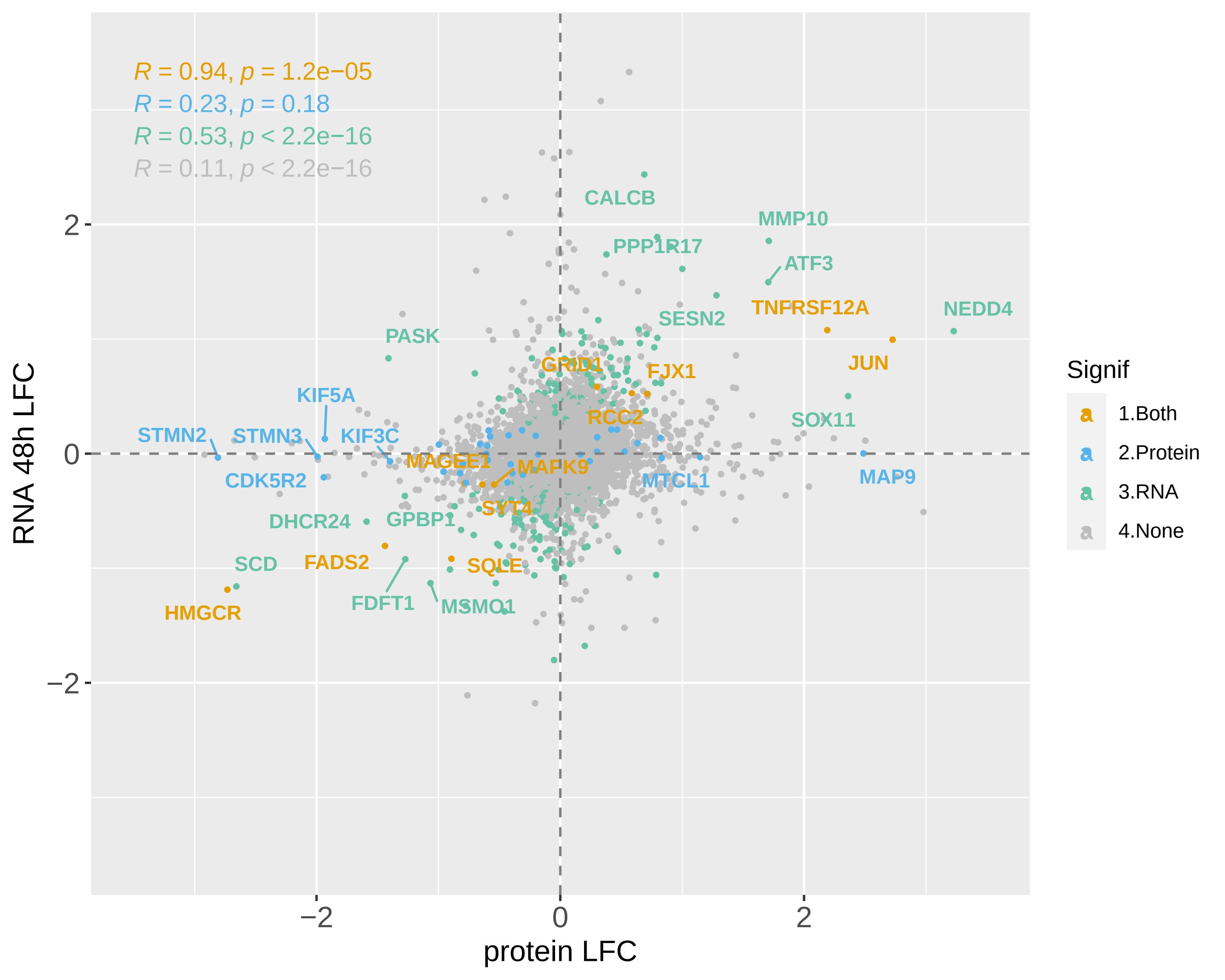

R = 0.94, p = 1.2e−05
R = 0.23, p = 0.18
R = 0.53, p < 2.2e−16
R = 0.11, p < 2.2e−16
CALCB
MMP10
PPP1R17
ATF3
TNFRSF12A
NEDD4
SESN2
PASK
JUN
GRID1
FJX1
KIF5A
RCC2
SOX11
STMN2
STMN3
KIF3C
MAGEE1
MAPK9
CDK5R2
MTCL1
MAP9
SYT4
DHCR24
GPBP1
SCD
FADS2
SQLE
HMGCR
FDFT1
MSMO1
2
0
−2
−2
0
2
RNA 48h LFC
protein LFC
Signif
1.Both
2.Protein
3.RNA
4.None
